# Comparison of DNA vaccines with AddaS03 as an adjuvant and an mRNA vaccine against SARS-CoV-2

**DOI:** 10.1016/j.isci.2024.110969

**Published:** 2024-10-15

**Authors:** Praveen Neeli, Dafei Chai, Xu Wang, Navid Sobhani, George Udeani, Yong Li

## Main text

(iScience *26*, 107120; July 21, 2023)

In the previously published version of the paper, the term “AS03” was used to describe the AddaS03 adjuvant used in animal experiments. This could lead to confusion among the trade and public as to a connection between the AddaS03 adjuvant and GSK’s AS03. The authors clarify that no AS03 from GSK was used in this study, and the results obtained with AddaS03 are not transposable to the GSK’s AS03 adjuvant. The article has now been corrected, and the conclusions of this paper remain unchanged. The authors apologize for any confusion caused.

**Figure:**
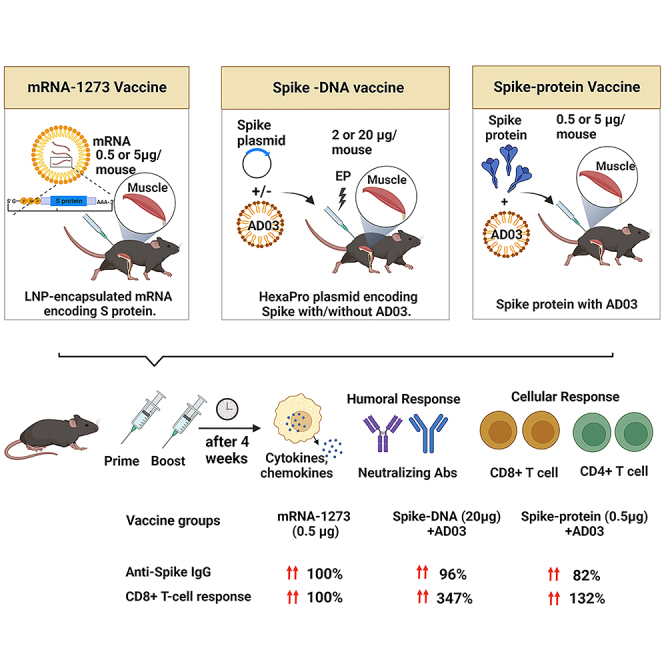
Graphical abstract (corrected)

**Figure:**
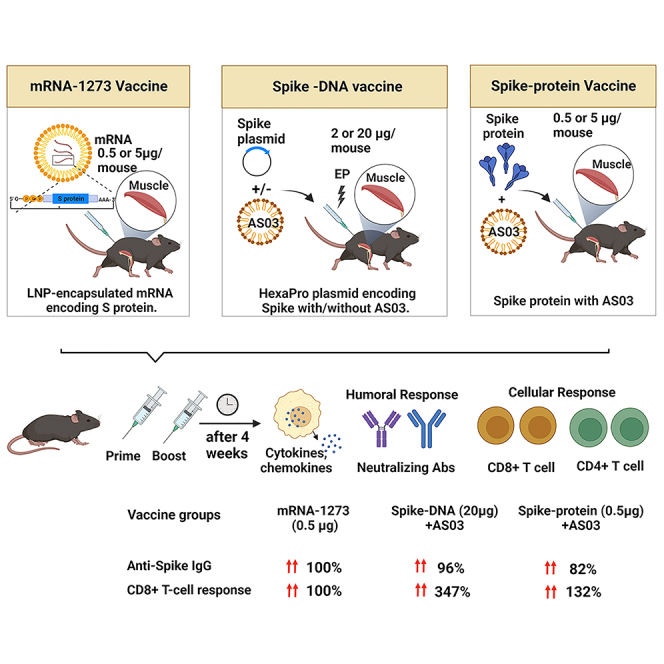
Graphical abstract (original)

**Figure:**
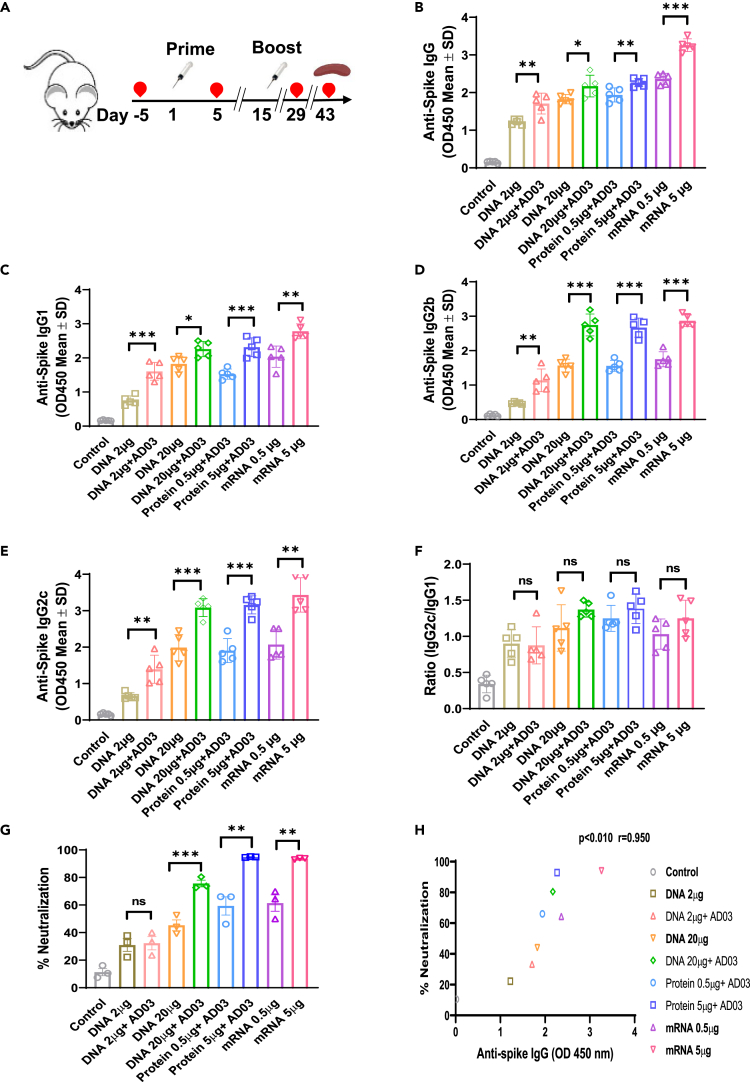
Figure 2. Spike-DNA vaccination induces a potent humoral response in immunized mice (corrected)

**Figure:**
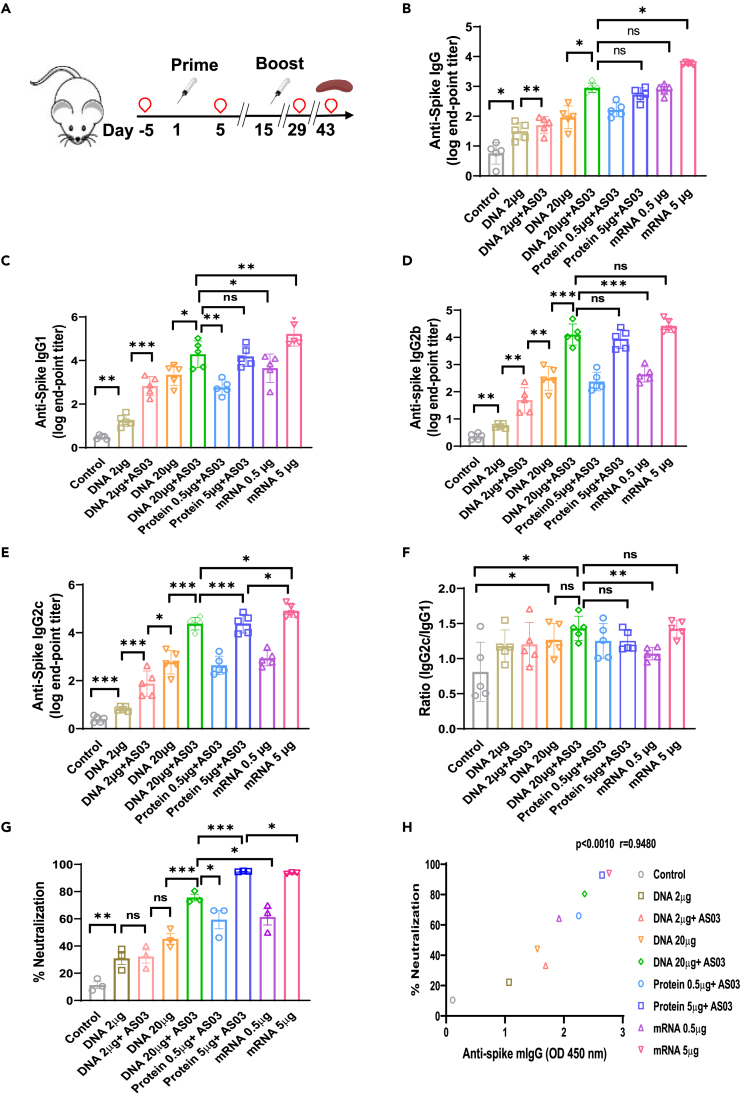
Figure 2. Spike-DNA vaccination induces a potent humoral response in immunized mice (original)

**Figure:**
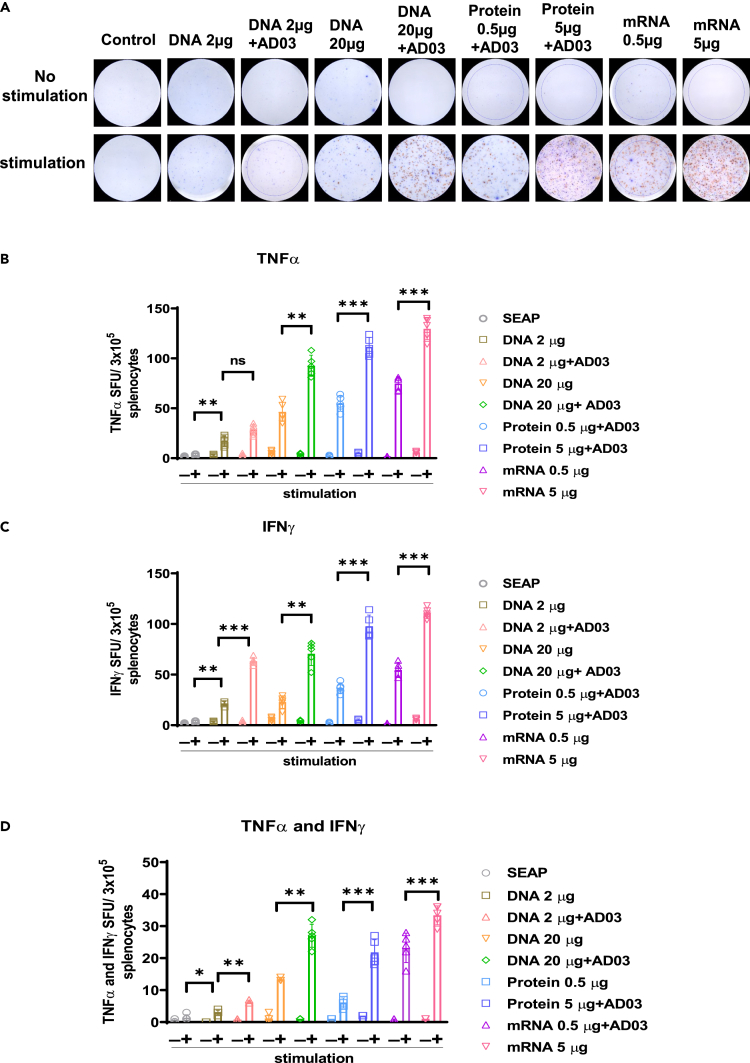
Figure 3. Spike-DNA vaccine potentiates functional T cells specific to SARS-CoV-2 Spike protein (corrected)

**Figure:**
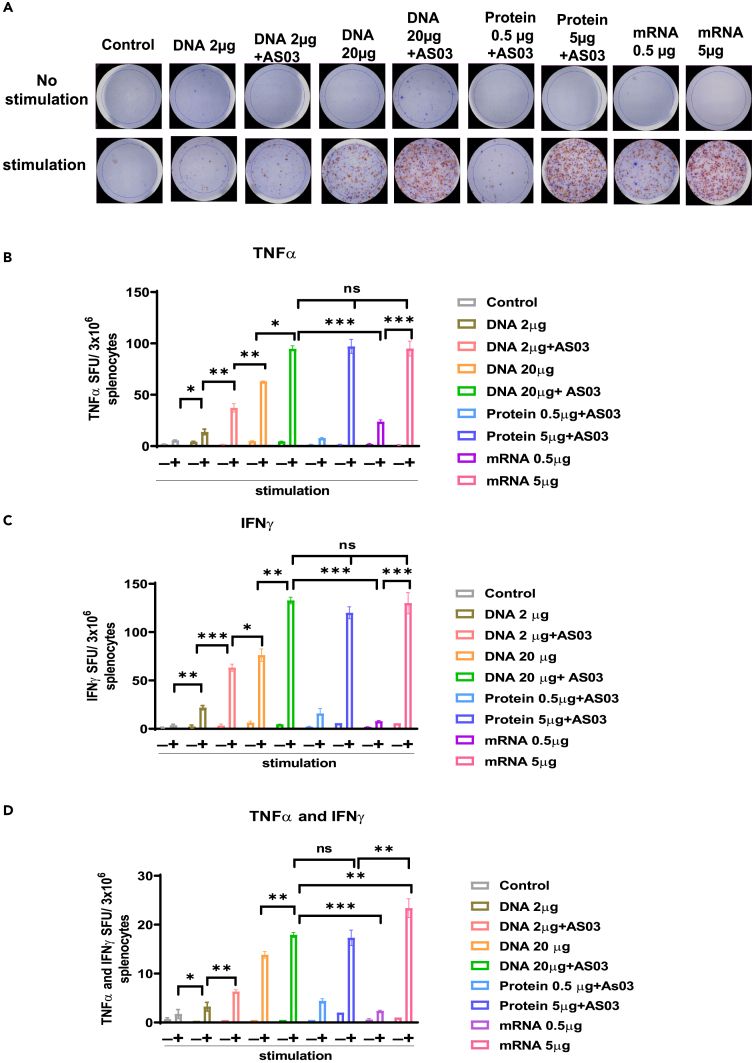
Figure 3. Spike-DNA vaccine potentiates functional T cells specific to SARS-CoV-2 Spike protein (original)

**Figure:**
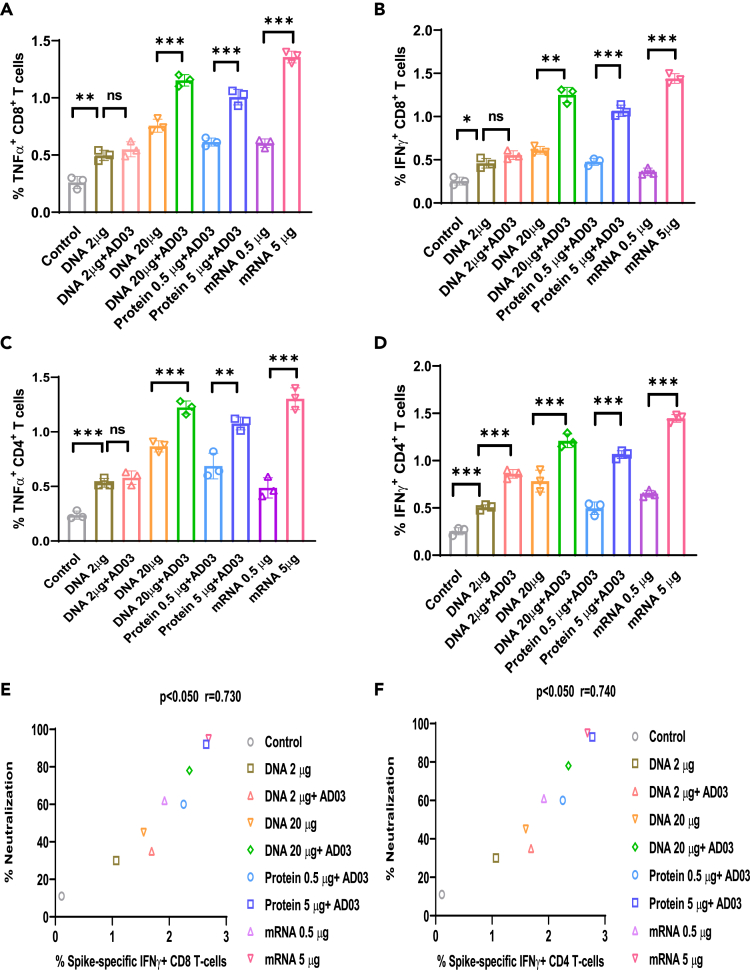
Figure 4. Spike-DNA vaccine induces CD8^+^ and CD4^+^ T cell responses specific to SARS-CoV-2 Spike protein (corrected)

**Figure:**
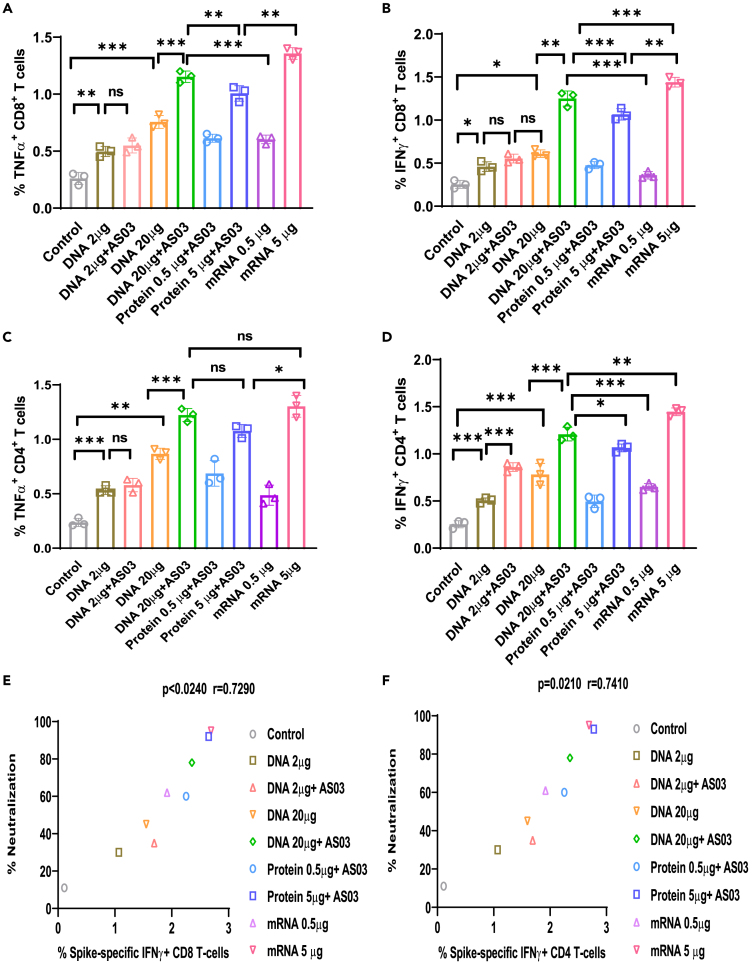
Figure 4. Spike-DNA vaccine induces CD8^+^ and CD4^+^ T cell responses specific to SARS-CoV-2 Spike protein (original)

**Figure:**
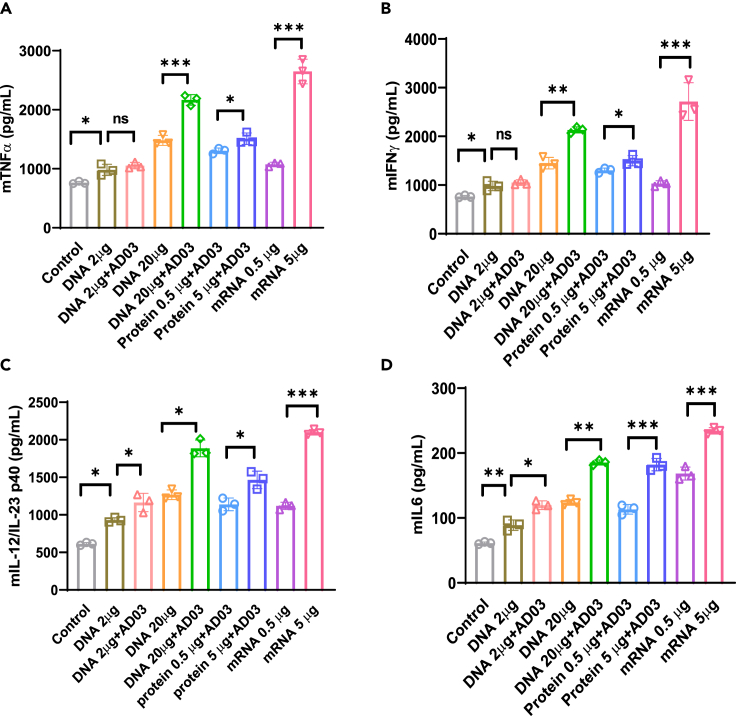
Figure 5. Spike-DNA vaccination induces proinflammatory cytokine production in Spike-stimulated splenocytes (corrected)

**Figure:**
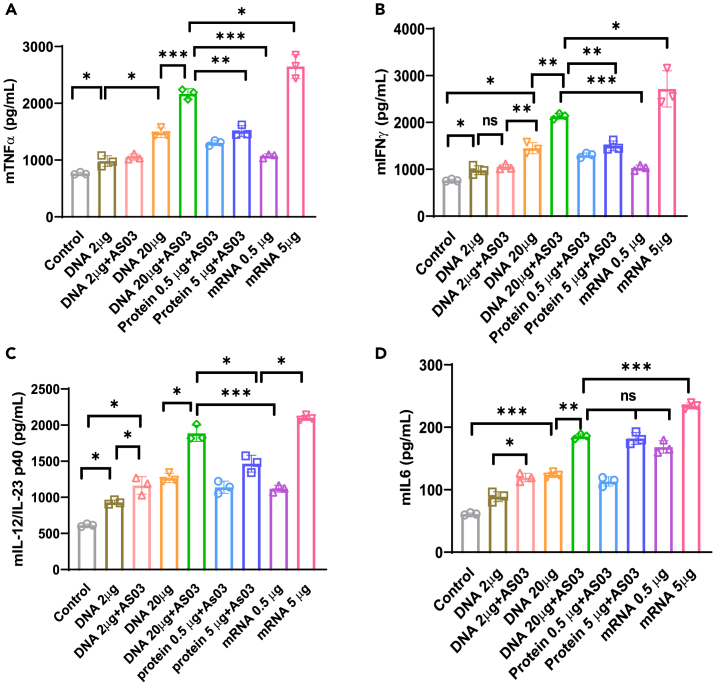
Figure 5. Spike-DNA vaccination induces proinflammatory cytokine production in Spike-stimulated splenocytes (original)

**Figure:**
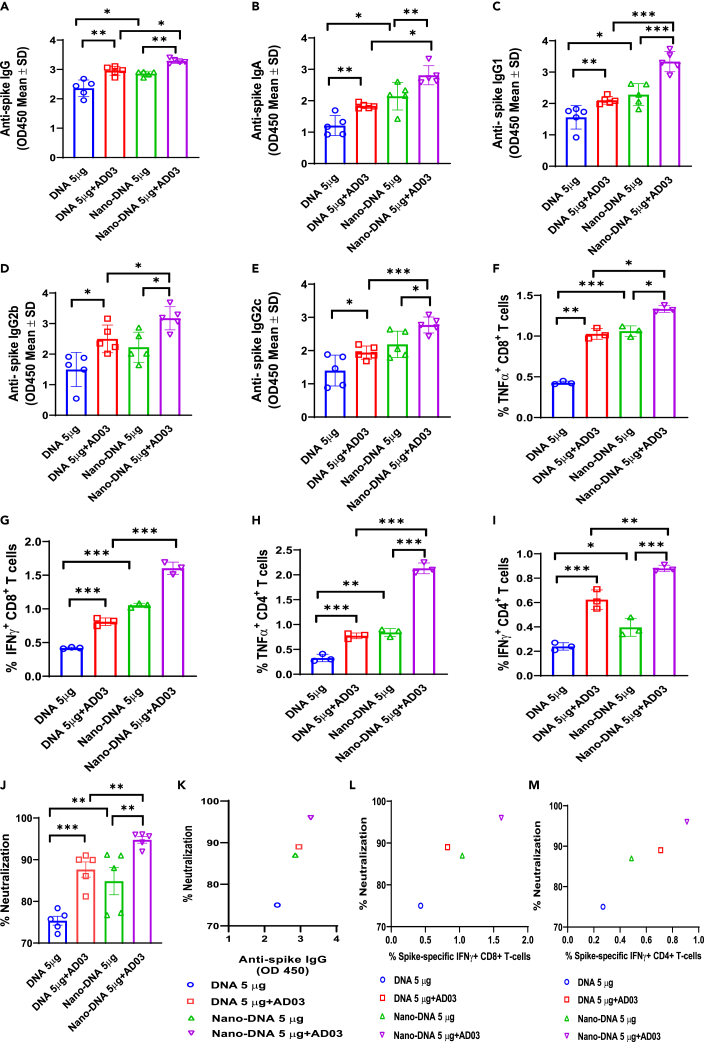
Figure 6. Mice immunized with Nano-DNA exhibit higher IgG and T cell responses than conventional plasmid; Adjuvant AS03 potentiates immune responses (corrected)

**Figure:**
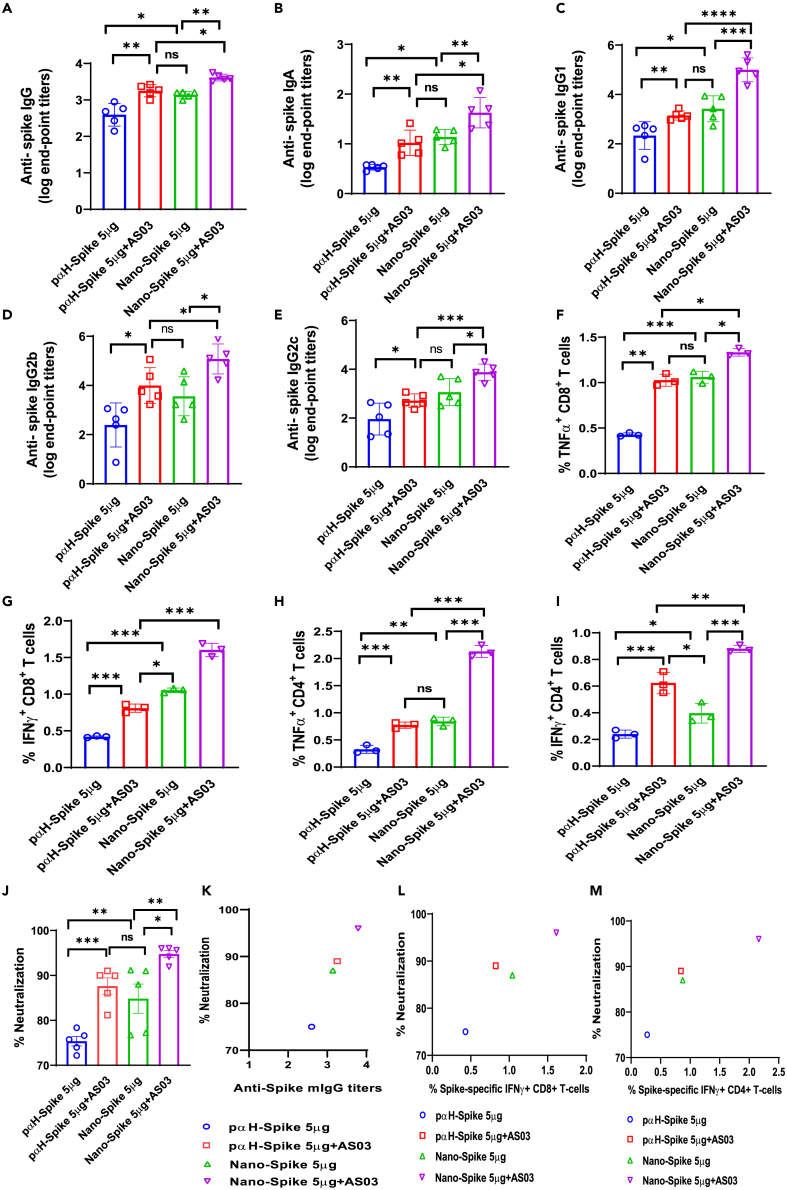
Figure 6. Mice immunized with Nano-DNA exhibit higher IgG and T cell responses than conventional plasmid; Adjuvant AS03 potentiates immune responses (original)

**Figure:**
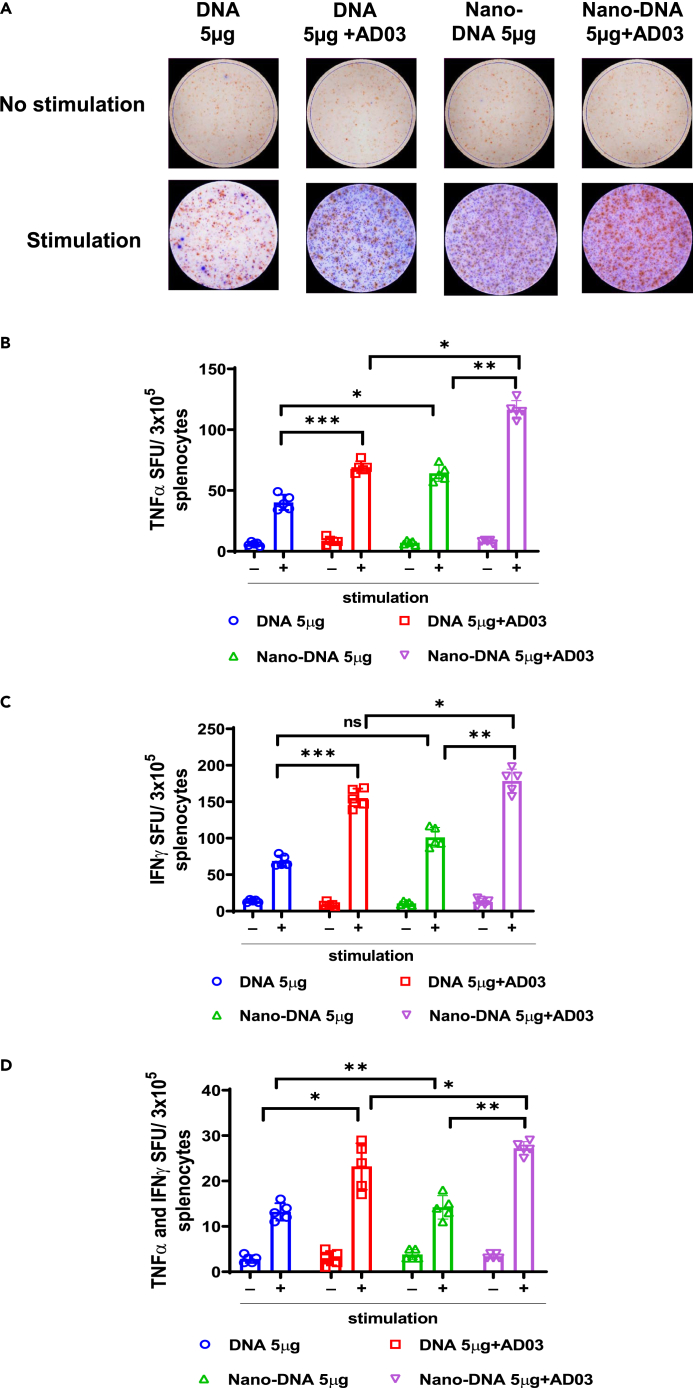
Figure 7. Mice immunized with Nano-DNA potentiate functional T cells specific to SARS-CoV-2 Spike compared to the conventional plasmid (corrected)

**Figure:**
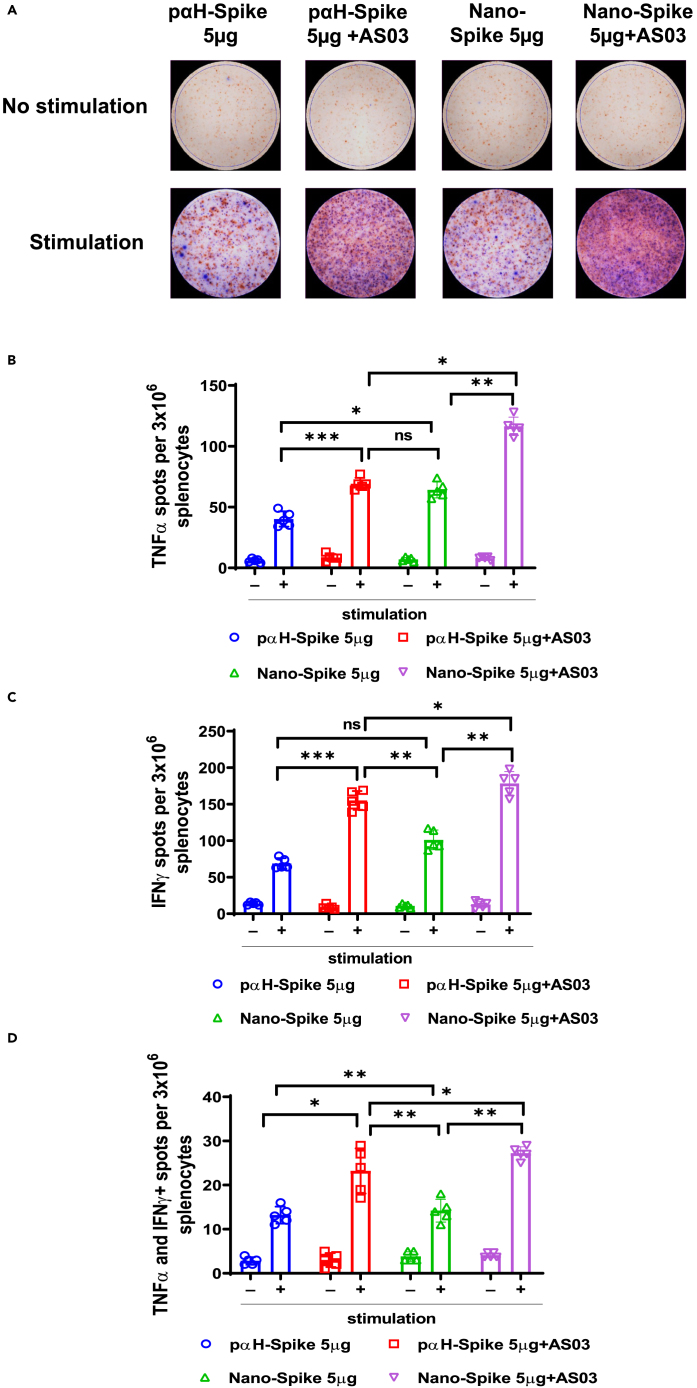
Figure 7. Mice immunized with Nano-DNA potentiate functional T cells specific to SARS-CoV-2 Spike compared to the conventional plasmid (original)

